# Effect of herbal formulation intake on health indices in albino Wistar rat model

**DOI:** 10.1002/fsn3.2009

**Published:** 2020-11-10

**Authors:** Adila Naseem, Saeed Akhtar, Muhammad Faisal Manzoor, Aysha Sameen, Anam Layla, Khurram Afzal, Emad Karrar, Abdul Rahaman, Tariq Ismail, Nazir Ahmad, Azhari Siddeeg

**Affiliations:** ^1^ Institute of Food Science & Nutrition Bahauddin Zakariya University Multan Pakistan; ^2^ School of Food Science and Engineering South China University of Technology Guangzhou China; ^3^ School of Food and Biological Engineering Jiangsu University Zhenjiang China; ^4^ National Institute of Food Science & Technology University of Agriculture Faisalabad Faisalabad Pakistan; ^5^ State Key Laboratory of Food Science and Technology School of Food Science and Technology Jiangnan University Wuxi China; ^6^ Institute of Food & Home Sciences Government College University Faisalabad Pakistan; ^7^ Department of Food Engineering Faculty of Engineering University of Gezira Wad Medani Sudan

**Keywords:** bilirubin, hypercholesterolemia, low‐density lipoprotein, toxicity, traditional medicine

## Abstract

Dyslipidemia management activity of ginger‐, garlic‐, and lemon‐based herbal mixture was tested as paste and herbal extract in hypercholesterolemic adult male albino rats. Atherogenic diet‐induced hypercholesterolemia in rats was treated by supplementing the diet with 2.5% herbal paste (4.2 g/kg b.w.) or 2.5 ml oral gavage (20 ml/kg b.w.) of liquid herbal extract daily for 42 days. Hematological and serological outcomes of herbal formulation feeding were compared with the cholesterol‐fed positive control and normal control. The results suggest the significant (*p* < .05) inhibitory properties of herbal paste and liquid extracts against dyslipidemia showing 31%–37%, 62%–68%, and 40%–56% lower levels of total cholesterol, triglycerides (TGs), and low‐density lipoprotein cholesterol (LDL‐C), respectively. Treating cholesterol‐fed animals with herbal paste and extract significantly (*p* < .05) increased total protein (5–5.5 g/dl) and serum albumin (3.7–4.2 g/dl) concentration as compared to the normal control. Contrary to significant hypocholesterolemic activity, higher serum total bilirubin levels, that is, 0.70 mg/dl, were observed in rats subchronically exposed to herbal paste and liquid extracts. Nonsignificant (*p* > .05) impact of herbal formula feeding was observed on hematological indices except lymphocyte counts, that is, 93% in rats fed on herbal paste. The results validate conventional hypocholesterolemic claims associated with ginger‐, garlic‐, and lemon‐based herbal formulations; however, deeper insight into their dose‐dependent response in hypercholesterolemia is necessitated to rule out the toxicological impact on the consumer.

## Graphical abstract

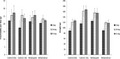

## 1 INTRODUCTION

Global Health Observatory data declare elevated cholesterol as an important risk factor in ischemic heart disease while its role in mortality index is significantly high with an annual mortality count of 2.6 million and 29.7 million disability‐adjusted life years (DALYs) (World Health Organization, 2018). Abnormal increment in serum triglycerides and total cholesterol had been documented to anticipate constriction or otherwise obstruction of the vessels predominately the heart vessels (Senior et al., 2019). Increased blood cholesterol levels in the blood are not a sickness; in fact, it is a disturbance in the balance of lipid metabolites, which may trigger the onset of certain health disorders (Richards et al., 2017). The death rate due to heart diseases has grown up in the last 20 years, and public health experts relate increased morbidity and mortality burden of cardiovascular disorders with hypercholesterolemia as a major risk factor among some others (Gupta et al., 2016). Low‐density lipoprotein (LDL) is suggested as an approved marker for coronary heart diseases and is considered as a risk factor for cerebral infarction and myocardial infarction as well (Navar‐Boggan et al., 2015).

Herbal formulations are a traditional system for the treatment and management of various ailments such as atherosclerosis, diabetes, hemorrhage, and inflammatory disorders (Rastogi et al., 2016). Intact herbs, their extracts, and essential oils had been extensively explored as an ethnopharmacological approach to measure their effectiveness against various diseases such as atherosclerosis, diabetes, hemorrhage, and inflammatory disorders. Herbal formulations are in practice therapeutics in various traditions for the treatment and prevention of hyperlipidemia. Such formulations are derivatives of plant‐based ingredients or bioactive compounds with least to no risk of toxicity to the human when compared with their synthetic analogs that constitute approximately 70% of the modern age medicine supplies (Verma & Singh, 2008).

Therapeutic properties of herbs and spices such as mint, onion, coriander, ginger, okra, cumin, lemon, garlic, and apple cider have been documented against obesity, hypercholesterolemia, hypertension, and cardiovascular disorders (Akhtar et al., 2019; Manzoor et al., 2019; Yasin et al., 2020). Garlic is known to be a primitive food with lipid‐modulating properties for ages (Pathmanathan et al., 2010). Apple cider vinegar has also gained researchers' attention since the past few decades due to its lipid‐mediating properties (Fratianni et al., 2007). Honey has also been cited to mediate dyslipidemia in addition to its therapeutic role in surgical debridement, decubitus, burns, infantile gastroenteritis, and hepatitis A (Khalil et al., 2010). Ginger is widely used in Ayurveda, Unani, and Chinese medicine as an ethnic remedy against inflammatory and gut disorders. Alike, ginger powder and its extracts have been reported efficacious in reducing total cholesterol, low‐density lipoprotein (LDL) cholesterol, and the degree of atherosclerosis (Bolanle, 2011).

Refer to their wide acceptability as a natural remedy for various health ailments, ginger and lemon are commercially exploited as ethnopharmacological natural drugs (Meo et al., 2017). Garlic has been used as a seasoning agent; however, it has a broad application in ethnopharmacology as an ethnic hypercholesterolemic drug. A sizable number of scientific investigations confirmed the hypolipidemic effect of the garlic (Sahebkar et al., 2016). An appreciable amount of scientific data are available which validate the significance of garlic constituents and its products (i.e., extracts and paste) which may prevent an increase in LDL cholesterol concentration, and reduce cholesterol synthesis by inhibiting 3‐hydroxy‐3‐methyl‐glutaryl (HMG) CoA reductase enzyme production (Ajayi & Ajayi, 2014). Garlic contains numerous health constituents of primary and secondary nature including fructan, essential oil, prostaglandins, anthocyanins, pectin, nicotinic acid, lectin, flavonoids, adenosine, vitamins B1, B_2_, B_6_, C, and E, glycolipids, biotin, essential amino acids, phospholipids, and fatty acids and steroid glycosides, S‐allylcysteine, and selenium which work in synergy as antioxidants, energy release, disease prevention, and reducing the cholesterol synthesis with various other health benefits (Gyu‐Bae et al., 2012; Rehman et al., 2020). Previous studies on animal models have reported garlic as a carrier of antiatherogenic, antithrombotic, antilipidemic, antihypertensive, and antiglycemic compounds (You et al., 2009).

In the backdrop of extended application of commonly consumed kitchen herbs in preventing and treating dyslipidemia, this study was planned to investigate the nutraceutical potential of a conventional herbal formulation against dietary‐induced hypercholesterolemia. Further, the study was aimed at exploring toxicological responses of consuming local herbal formulation on various physiological indices.

## 2 MATERIAL AND METHODS

### 2.1 Procurement of raw material, chemicals, and reagents

The study was conducted in the animal‐rearing facility of the Institute of Food Science and Nutrition after approval from the Bioethics Committee of the Faculty of Agricultural Sciences & Technology, Bahauddin Zakariya University, Multan. Fresh lemon, ginger, garlic, apple cider vinegar, and honey were procured from the local market of Multan, Pakistan. All reagents, standards, and chemicals were of analytical grade and purchased from the local distributors of Sigma‐Aldrich (Sigma‐Aldrich) and Merck (Merck KGaA). Diagnostic kits used in serological testing were purchased from the local distributors of Cayman Chemicals (Cayman Europe) and Sigma‐Aldrich, Bioassay (Bioassay Chemical Co.).

### 2.2 Sample preparation and extraction

Postprocurement screening and washing of ginger, garlic, and lemon were performed to ensure their wholesomeness, and washing was performed with potable water to remove adherent materials. Fresh herbs and lemon were ground to obtain either the paste or the fresh juice. Homogenously ginger, garlic, and lemon (processed in equal amount, i.e., 20%, *w/w*) were ground and mixed with apple cider vinegar and honey in the same proportion to generate an herbal paste. Raw juices of lemon, ginger, and garlic were extracted and filtered from the Whatman filter paper no. 41. Herbal extracts were mixed with apple cider vinegar and honey in equal proportion (20%, v/v). Both herbal preparations were stored at refrigeration temperature (6 ± 2°C) for further use.

### 2.3 Experimental protocol

Twenty male albino Wistar rats were procured from the laboratory animal‐rearing facility of the faculty of Pharmacy, Bahauddin Zakariya University, Multan, Pakistan. The average weight of randomly selected male rats was in the range between 120 and 130 g in all groups. Animal‐rearing facility conditions were optimized for the light–dark cycle, that is, twelve‐hour day and night cycle, temperature (22 ± 5°C), and humidity (55%–57%). Before initiate an experimental diet, rats were acclimatized to the new environment and access to the control diet and water ad libitum for 1 week. After the acclimatization period, rats were divided into four experimental groups, that is, T_NC_, T_PC_, T_1,_ and T_2_ namely negative control, positive control, herbal extract‐fed, and herbal paste‐fed groups, respectively, each containing four rats. The basal diet of the rats was prepared following the laboratory animal diet guidelines suggested by Levrat‐Verny et al. (1999). Treatment diets were prepared by incorporating 2.5 g and 2.5 ml of herbal paste and herbal extracts, respectively (Table 1). Rats were monitored for water and feed intake regularly while weight measurement was scheduled weekly during the feeding trial.

**TABLE 1:** Composition of control and experimental diets for albino Wistar rats (g/100 g)

Ingredients	T_NC_	T_PC_	T_1_	T_2_
Corn starch	65.5	65.5	65.5	65.5
Casein	10	10	10	10
Cellulose	10.5	10.5	10.5	10.5
Corn oil	10	9	9	9
Mineral mixture	3	3	3	3
Vitamin mixture	1	1	1	1
Herbal extracts (O)	0	0	2.5	0
Herbal paste (Hb)	0	0	0	2.5
Cholesterol	0	1	1	1

Note

Control (−ve) is normal control. Control (+ve) is cholesterol‐fed.

### 2.4 Hematological and serological sampling

Random sampling was performed from the control and treatment groups on the 0th and 42nd days of the study. Rats were restricted from the diet 8 hr before anesthesia and sampling. Assorted blood was drawn by cardiac puncturing, and animals were sacrificed humanely by cervical dislocation. Hematological analysis samples were collected in labeled vacutainers containing 4% ethylenediaminetetraacetic acid (EDTA) solution. Samples for serological analysis were collected in clot activator vacutainers and centrifuged at 12,000 rpm for 3 min to separate the serum from plasma, and were stored in an ultra‐freezer at −79°C for further laboratory examination.

### 2.5 Biochemical analysis

Serum collected was examined for total cholesterol, triglycerides (TAG), high‐density lipoprotein (HDL), serum uric acid, urea, plasma glucose, total protein, albumin, creatinine, bilirubin, and serum enzyme level, that is, serum glutamate pyruvate transaminase (SGPT) and serum glutamate oxaloacetate transaminase (SGOT), by using diagnostic kits following the procedures followed by Olubanke (2012). Hematological analysis including red blood cells (RBCs), white blood cells (WBCs), hematocrit concentration, mean corpuscular concentration, hemoglobin, platelets, neutrophils, mean corpuscular volume, and lymphocytes were measured on hematological analyzer following the procedure as mentioned by Dacie and Lewis (1984).

### 2.6 Statistical analysis

All analyses were performed in triplicate. The data were statistically analyzed using a two‐way analysis of variance (ANOVA) technique. Mean comparisons were performed using the least significant difference, and the level of significance was set as *p* < .05.

## 3 RESULT AND DISCUSSION

### 3.1 Effect on feed intake and body weight of the rats

**FIGURE 1:**
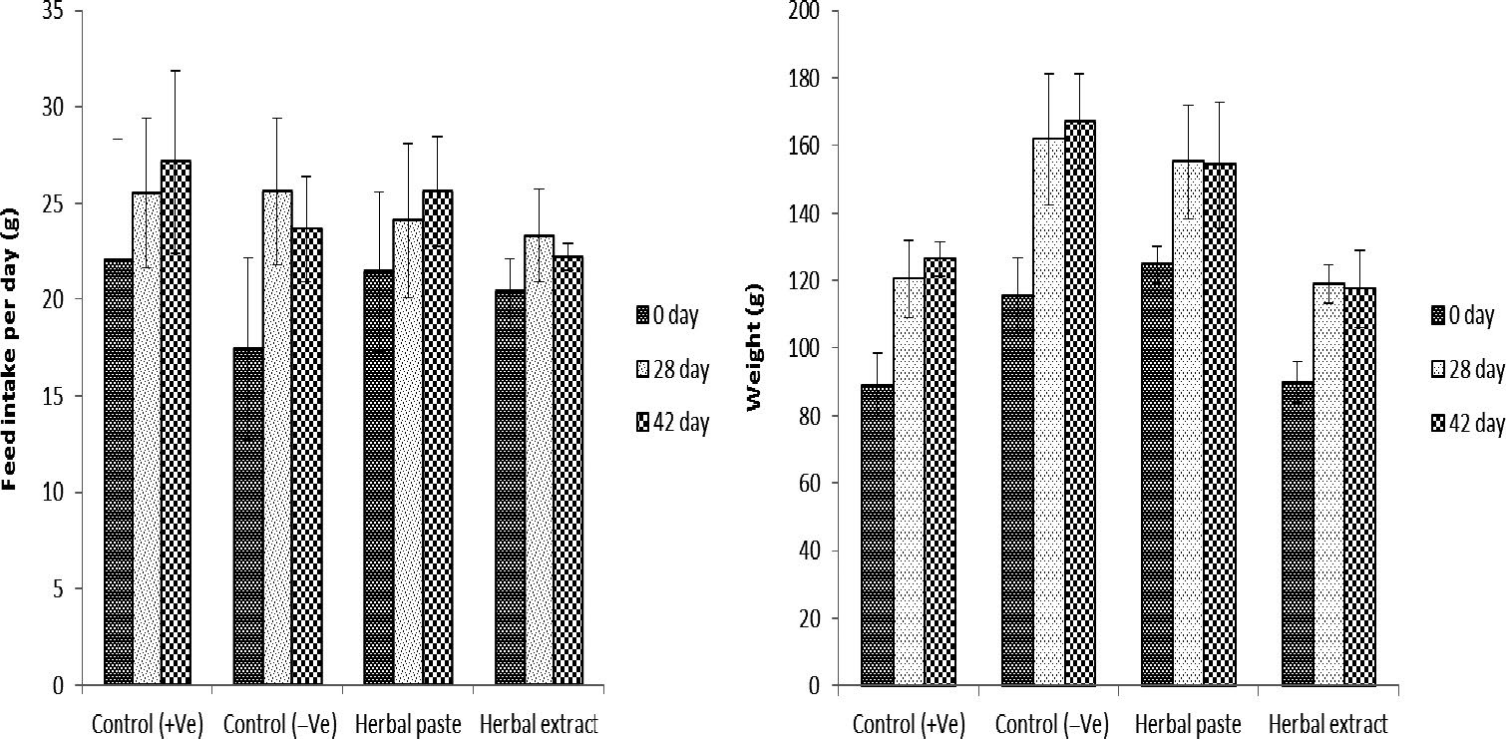
Effect of herbal extracts and paste feeding on mean dietary intake and weight gain proportion during a 42‐day feeding period

Feed intake data from the normal and treated rats' model is presented in Figure 1. The results suggest a nonsignificant (*p > .05*) effect of type of herbal formula feeding and feeding duration on feed intake trends; however, feeding herbal formula to hypercholesterolemic rats' model anticipated significant (*p* < .01) increment in basal weight at 42nd day of formula feeding (Figure 1). The weight gain trend was observed highest in positive control and herbal paste‐fed hypercholesterolemic rats, that is, 116–167 g and 125–155 g, respectively, and was nonsignificantly (*p* < .05) different from each other at 42nd day of feeding. Oral herbal decoction‐fed rats' group was presented with the lowest weight gain trend when compared with the positive control and herbal paste‐fed rats' groups, and the mean weight of the treated hypercholesterolemic rats was 126 g that was increased 50% to that of the first weight while ~75% weight gain from the initial weight was observed in herbal paste‐fed rats.

The outcomes for feed intake of the hypercholesterolemic rats of the present study are in close resemblance with the findings of Awan et al. (2019) wherein a nonsignificant difference in feed intake (20.28–20.8 g/rat per day) was observed among rats fed on the natural herbal extract and the normal control during 42‐day feeding trial. Previously, Kiasalari et al. (2009) had also reported the nonsignificant impact of feeding herbal decoction to hypercholesterolemic rats on feed intake. Contrary to these reports and our findings, significant (*p* < .05) improvement in feed intake by rats fed on the herbal mixture and supercritical garlic extracts was reported by Awan et al. (2019) and Naseem et al. (2016) respectively, suggesting bioactive fractions of the formula‐fed to enhance satiety function when compared with the normal control. Our results, however, demonstrated normal appetite functioning in herbal paste formula feeding with comparable feed intake response as that of the normal control rats.

The results for the weight gain trends of herbal decoction‐fed hypercholesterolemic rats in the present study were contradictory to the findings of Piao et al. (2013) wherein the weight of rats was significantly (*p* < .05) increased on feeding the herbal decoction. This significant (*p* < .05) increase in weight of rats in the reported study may be suggestive of the fatty acid composition of the poly‐herbal formula, that is, containing higher amounts of saturated fats; however in our study, fresh juice of garlic‐, ginger‐, and lemon‐based herbal decoction on account of its trace fats residues did not anticipate any significant increase in calories of the fed formula. Earlier, a study by Kondo et al. (2009) has suggested bioactive compounds of natural herbal extracts as the dietary differential to enhance weight in experimental rats.

### 3.2 Effect on serological parameters of the rats

**FIGURE 2:**
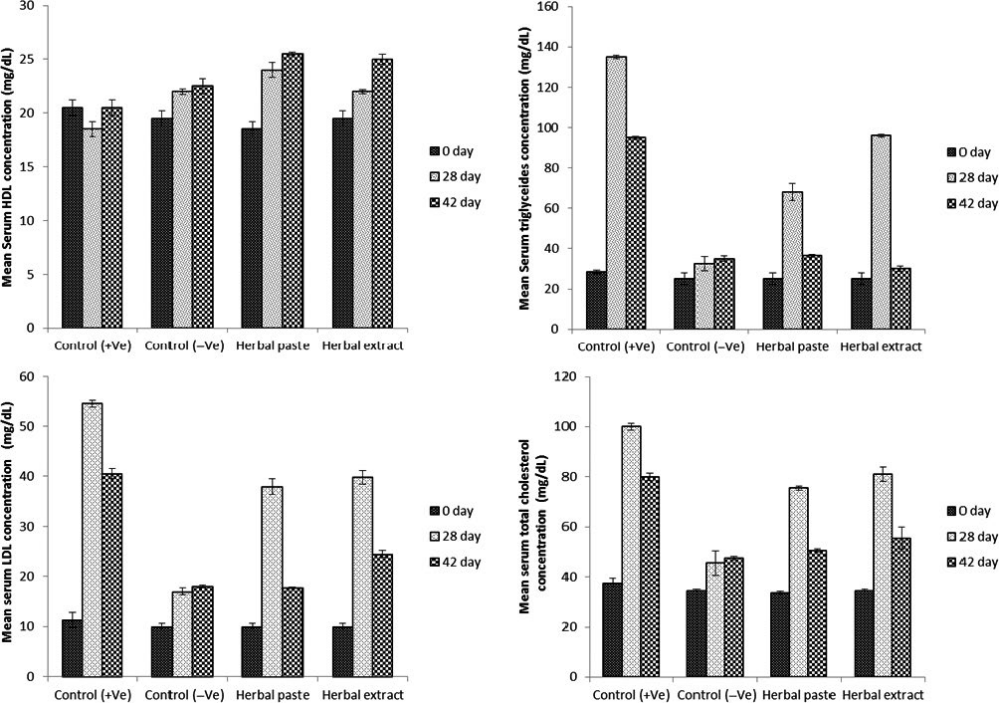
Effect of herbal extract and paste feeding on lipid profile of hypercholesterolemic rats during a feeding duration of 42 days

Despite the tremendous acceptability of plant‐based alternate and complementary formulations for the treatment of dyslipidemia in various cultures, the synergistic effects of different herbs used in on formulation for the treatment of various health ailments and their possible side effects have been least explored. Refer to the findings of our study wherein conventional herbal formulations were tested for their possible ameliorative and or toxicological effect on the hypercholesterolemic rat model, the study suggests a significant difference (*p* < .01) in total cholesterol of the positive control (hypercholesterolemic, nontreated rats) and herbal formulation‐fed hypercholesterolemic rats while nonsignificant differences (*p* > .05) in total cholesterol concentrations were observed among negative control (normal rats), and herbal paste‐ and herbal decoction‐fed hypercholesterolemic rats (Figure 2; Table 2). The identical response of feeding 2.5% herbal paste (*w/w*) and 2.5% herbal oral solution (*v/w*) was noticed for HDL cholesterol while feeding herbal paste and oral decoction to hypercholesterolemic rats significantly reduced total cholesterol, triglycerides, and LDL cholesterol, that is, 36%–40%, 6%–67%, and 30%–33%, respectively. HDL cholesterol was lowest in positive control rats (20.5 mg/dl), and maximum serum HDL‐C was found in the herbal paste‐fed rats group followed by oral decoction‐fed group and negative control, that is, 25.5, 25, and 22.5 mg/dl, respectively.

**TABLE 2:** Effect of paste and extract on serological parameters of a hypercholesterolemic rat model

Parameter	Control (−ve)	Control (+ve)	Herbal paste	Herbal extract
Total protein	3.00^b^	3.75^b^	5.50^a^	5.00^a^
Albumin	2.20^b^	3.20^ab^	4.20^a^	3.70^a^
Creatinine	0.50	0.45	0.40	0.50
Bilirubin	0.25^b^	0.30^b^	0.70^a^	0.70^a^
ALT	55.00	56.50	58.00	5.08
AST	48.50	37.00	46.00	35.50
Urea	24.00	24.00	19.50	26.50
Uric acid	5.90	6.10	6.75	6.50
Glucose	192.5	204.0	196.50	178.0
Total cholesterol	47.50^b^	80.00^a^	50.50^b^	55.50^b^
Triglycerides	35.00^b^	95.00^a^	36.50^b^	30.00^b^
HDL	22.50^b^	20.50^c^	25.50^a^	25.00^a^
LDL	18.00^b^	40.50^a^	17.70^b^	24.50^b^

Note

Values sharing same lettering in a row are not significantly different from each other at *p* < .05, while means in a row with no lettering are not significantly different from each other. Control (−ve) is the normal control. Control (+ve) is cholesterol‐fed. Herbal paste is fed on 2.5% herbs paste. Herbal extract is fed on 2.5 ml herbal juice.

Feeding herbal paste and herbal decoction to hypercholesterolemic rats for 42 days resulted in significantly (*p* < .05) higher levels of total protein, that is, 5–5.5 mg/dl, when compared with the positive and normal control groups that were observed with relatively lower serum total protein contents, that is, 3–3.75 mg/dl (Table 2). Serum albumin concentration in rats fed on herbal paste was highest, that is, 4.2 mg/dl among the treated rats' groups followed by herbal decoction‐fed rats' (3.7 mg/dl), positive control (3.2 mg/dl), and negative control (2.2 mg/dl) groups. The identical response of feeding herbal formulation to hypercholesterolemic rats was observed on bilirubin levels. Feeding herbal decoction to hypercholesterolemic rats for 42 days significantly (*p* < .05) increased bilirubin contents up to 0.7 mg/dl while serum bilirubin concentration in the same study period was 0.2–0.3 mg/dl in normal control rats. Nonsignificant (*p* > .05) difference in serum creatinine, alanine transaminase (ALT), aspartate transaminase (AST), urea, uric acid, and glucose contents were observed in herbal formulation‐fed hypercholesterolemic rats when compared with the normal control (Table 2), thus suggesting nontoxicological response of the conventional formulations on various serological parameters at maximum levels of supplementation.

The results on the combined effect of ginger‐, garlic‐, lemon‐, apple cider vinegar‐, and honey‐based formulations on serological parameters of hypercholesterolemic rats are in close resemblance to the findings of Awan et al. (2019) and Uddin et al. (2016)wherein garlic paste, ginger juice, and polyphenol‐rich garlic extracts were individually reported efficacious in treating dyslipidemia. Another study by Gardner et al. (2001) revealed a feeding dehydrated blend of garlic powder at 500–1,000 mg/day for 12 weeks significantly (*p* < .05) reduced LDL‐C with statistically significant (*p* < .05) increment in HDL‐C levels in human subjects. In another study, feeding garlic decoctions to hypercholesterolemic rats and human subjects reduced serum cholesterol by 7% and inhibited de novo synthesis of cholesterol (Yeh & Liu, 2001). In agreement with the findings of our study, Joo et al. (2013) also reported daily dietary intake of garlic extract to yield a significant (*p* < .05) reduction in serum LDL‐C concentration by 43%.

Hypocholesterolemic properties of garlic and ginger are attributed to their bioactive compounds and trace elements including diallyl sulfide (DAS), S‐acetylcysteine, allicin, *N*‐acetylcysteine (NAC), S‐allylcysteine (SAC), gingerols and its isomers, quercetin, zingerone, selenium, and tellurium (de Las Heras et al., 2017). Lipid‐modulating properties of garlic and ginger are associated with their inhibitory role on lipoprotein lipase, 3‐hydroxy‐3‐methoxyglutaryl coenzyme A (HMG‐COA) reductase, and increasing liver LDL receptor activity (Sumaira et al., 2012). In addition to LDL cholesterol‐reducing activity, feeding fresh garlic bulbs supplemented diet to the hypercholesterolemic rats was recorded to anticipate a significant (*p* < .05) increase in HDL‐C concentration up to 40% (Elmahdi et al., 2008). Naseem et al. (2016) reported a nontoxic response of herbal mixture matching in its composition to the herbal paste tested in this study. The results in line with the finding of our study suggested normal levels of serological indices including total protein, albumin, creatinine, ALT, AST, urea, and bilirubin in hypercholesterolemic rats fed on the herbal mixture.

### 3.3 Effect on hematological parameters of the rats

The toxicological effects of herbal medicine on abnormal laboratory values like those of hematological indices indicate altered physiology (Dasgupta, 2003). Dietary administration of 2.5% herbal paste and 2.5 ml herbal decoction to hypercholesterolemic rats did not significantly change hematological parameters including hemoglobin (HGB), monocytes, white blood cells (WBC), red blood cells (RBC), platelets, neutrophils, mean corpuscular volume (MCV), mean corpuscular hemoglobin concentration (MCHC), mean corpuscular hemoglobin (MCH) eosinophil, and hematocrit (HCT) excepting lymphocytes (Table 3). In comparison with the normal control rats, a moderate increase in lymphocyte counts was observed in hypercholesterolemic rats fed on herbal paste during a feeding duration of 42 days. Although none of the rats in the treatment and control groups breached normal levels of lymphocytes, moderate increment in lymphocytes may be suggestive of herbal paste ability to improve the immunological response of the dyslipidemia rats in stress. A significant increase in lymphocyte counts of dyslipidemia rats fed on guava extracts was reported by Kullu et al. (2013) as an immunological response against dyslipidemia. Nonsignificant (*p* > .05) differences in the count of white blood cells and red blood cells were reported by Naseem et al. (2016) in their study on hyperlipidemic rats fed on a poly‐herbal mixture. Contrary to the results of this study, Iranloye (2002) reported significant (*p* < .05) increase in hemoglobin concentration, white blood cell, red blood cell, neutrophil, monocyte, and lymphocyte counts of rats fed on garlic juice at 200 mg/kg during 30‐day feeding period. Low‐dose administration of aqueous extracts of ginger at 50 mg/kg was reported nontoxic in female rats in a study by Al‐Naqeeb et al. (2003), thus suggesting ginger extracts as quite safe at low levels. Feeding levels of herbal paste and decoction tested in this study were however lower (i.e., up to 170 µg/kg body weight) than reported in the abovementioned study.

**TABLE 3:** Effect of paste and extracts on hematological parameters of hypercholesterolemia rat model

Parameter	Control (−ve)	Control (+ve)	Herbal paste	Herbal extract
Lymphocytes	76.5^b^	76.5^b^	93.5^a^	83^b^
HGB	11.7	11.6	12.5	9.9
Monocytes	7.5	7.5	6	5
WBC	4.1	3.4	6.4	1.6
RBC	6.38	5.32	6.4	5.32
Platelets	398	398	293.5	255.5
Neutrophil	8.5	8.5	3	6.5
MCV	50.15	50.15	49.9	47.1
MCHC	40.3	40.3	39.8	39.05
MCH	20.3	20.2	19.9	18.4
Eosinophils	7.5	7.5	5.5	5.5
HCT	29	29	32.05	25.05

Note

Values sharing same lettering in a row are not significantly different from each other at *p* < .05, while means in a row with no lettering are not significantly different from each other. Control (−ve) is the normal control. Control (+Ve) is cholesterol‐fed. Herbal paste is fed on 2.5% herbs paste. Herbal extract is fed on 2.5 ml herbal juice.

## 4 CONCLUSION

Herbal medicines are primitive and conventional therapeutics for around 80% of the world's population due to their wide acceptability, availability, and affordability while their safety claims are in the process of validation in line with the consumer safety standards of the recent era. Findings of this subchronic exposure study on evaluating the synergistic effect of garlic‐, ginger‐, onion‐, apple cider vinegar‐, and honey‐based conventional herbal formulations against dyslipidemia validated existing therapeutic claims of the recipe. Contrary to their nontoxicological behavior against various hematological and serological markers, dietary exposure of herbal paste and liquid extracts at a rate of 4.2 g/kg b.w. and 20 ml/kg b.w., respectively, significantly increased serum bilirubin concentration of the rats. Likewise, higher lymphocyte counts were noticed in herbal paste‐fed treatment groups indicating a comparatively higher immune response. Although no mortality was observed in treatment groups; however, outcomes of the study warrant more careful toxicological assays to investigate safer consumption levels of the proposed formulation. Moreover, preliminary findings of this study further emphasize determining the immunological response of composite herbal formulations to rule out their suspected immunotoxicological effect.

## CONFLICT OF INTEREST

The authors declare no conflicts of interest.

## AUTHOR CONTRIBUTION

**Adila Naseem**: Formal analysis‐Equal. **Saeed Akhtar**: Conceptualization‐Equal. **Muhammad Faisal Manzoor**: Writing‐original draft‐Equal, Writing‐review & editing‐Lead. **Ayesha Sameen**: Data curation‐Equal, Software‐Equal. **Anam Layla**: Methodology‐Equal. **Khurram Afzal**: Data curation‐Equal, Software‐Equal. **Emad Karrar**: Data curation‐Equal, Software‐Equal. **Abdul Rahaman**: Writing‐review & editing‐Equal. **Tariq Ismail**: Conceptualization‐Equal, Supervision‐Equal, Writing‐original draft‐Equal. **Nazir Ahmad**: Data curation‐Equal, Writing‐original draft‐Equal. **Azhari Siddeeg**: Software‐Equal, Visualization‐Equal.

## Data Availability

The dataset supporting the conclusions of this article is included within the article.
